# Complete (Humoral and Cellular) Response to Vaccination against COVID-19 in a Group of Healthcare Workers-Assessment of Factors Affecting Immunogenicity

**DOI:** 10.3390/vaccines10050710

**Published:** 2022-04-30

**Authors:** Ewa Morgiel, Magdalena Szmyrka, Marta Madej, Agata Sebastian, Renata Sokolik, Iga Andrasiak, Maria Chodyra, Małgorzata Walas-Antoszek, Lucyna Korman, Jerzy Świerkot

**Affiliations:** 1 Department of Rheumatology and Internal Medicine, Faculty of Medicine, Wroclaw Medical University, 50-367 Wrocław, Poland; ewa.morgiel@umw.edu.pl (E.M.); magdalena.szmyrka@umw.edu.pl (M.S.); agata.sebastian@umw.edu.pl (A.S.); renata.sokolik@umw.edu.pl (R.S.); lucyna.korman@umw.edu.pl (L.K.); jerzy.swierkot@umw.edu.pl (J.Ś.); 2 Independent Researcher, 50-367 Wrocław, Poland; igaandrasiak@gmail.com; 3 Internal Medicine Department, Town Hospital Bolesławiec, 59-700 Bolesławiec, Poland; mmchodyra@gmail.com (M.C.); walasmalg@gmail.com (M.W.-A.)

**Keywords:** SARS-CoV-2 1, COVID-19 2, BNT162b2 3, vaccine 4, humoral immune response 5, cellular immune response 6

## Abstract

Vaccination is the best way to limit the extent of the COVID pandemic. Knowledge of the duration of the immune response will allow the planning of a vaccination protocol. This study aims to validate the complete (humoral and cellular) immune responses over time in large population groups following the full vaccination of healthcare professionals in real-life conditions and to assess the relationship between antibody levels and T-cell activity in relation to the characteristics of the study group. The samples for the study were obtained from volunteers (staff of two hospitals) on three occasions: before vaccination, T0, then 4–9 weeks after full vaccination (two doses BNT162b2), T1, and 7–9 months after vaccination, T2. The humoral response was investigated by the titre of anti-SARS-CoV-2 IgG antibodies to S1 protein. Assays were performed three times at intervals. The cellular response was assessed in a subgroup of 189 subjects by QuanT-Cell SARS-CoV-2 (IGRA). The assay was performed once. A group of 344 subjects fully vaccinated with the BNT162b2 vaccine were included in the study. The humoral response was observed in 100% of subjects at both 4–7 weeks and 7–9 months, but antibody titres fell by almost 90% in this interval. The cellular response was observed in 94% (177/189) of subjects 7–9 months after the second dose of vaccine. In subjects with a negative cellular response, eight out of 12 smoked. A factor associated with greater immunogenicity of vaccination was past SARS-CoV-2 infection. The administration of full BNT162b2 vaccination (two doses) induces humoral and cellular responses detectable even more than six months after vaccination. Smoking may be a factor associated with impaired cellular response to vaccination.

## 1. Introduction

The SARS-CoV-2 virus is the cause of a pandemic with many consequences, not only in terms of health, but also in the economic and social dimensions. Although in recent months the problem of SARS-CoV-2 seems to be easing, another wave of infections is still highly likely.

The humoral response following SARS-CoV-2 infection involves the production of antibodies directed against virus particle surface proteins located within the spike and nucleocapsid. The spike glycoprotein contains an S1 subunit containing a receptor-binding domain (RBD) involved in forming the binding of the virus to ACE2 receptor cells of the host. This is how the virus enters the cells. Antibodies neutralise the virus and block its binding to ACE2 receptors. Therefore, the level of antibodies directed to the spike protein appears to be a good indicator of the immune response after virus infection and after vaccination. It is also a biomarker of immunity [1]. 

T cells limit the spread of infection, remove infected cells, and protect against viral infection [2]. In patients with agammaglobulinaemia and other haematological disorders and those on immunosuppressive drugs with an impaired humoral response, the T cells have been attributed a protective function against viral infection. Individuals with past SARS-CoV-2 infection and after vaccination show virus-specific memory T cells. According to some researchers, if the virus slips through humoral protection, then the T-cell activity is the guarantee of a mild course of the disease [3]. However, in the light of current knowledge, it is difficult to determine the influence of cellular and humoral responses in protection against infection [2,4]. 

The first approved vaccine against SARS-CoV-2 was BNT162b2 (Pfizer-BioNTech) based on a novel technique. The vaccine contains genetic material (mRNA) encoding the full-length spike (S) protein and production of ‘vaccine’ antigens takes place in cells of vaccinated person. In clinical trials, protection against infection of more than 95% was provided in the first two months after vaccination, with reductions in hospitalisation and mortality from COVID-19 [5]. After vaccination, there is a gradual decrease in vaccine effectiveness and increased infections in the following months. However, it should be noted that the course of the disease is usually mild [6].

First, the persons in the so-called risk group had an opportunity to get vaccinated: healthcare workers and persons over 60. Full vaccination with the BNT162b2 vaccine consists of two doses 30 µg of the product. Full vaccination results in a high synthesis of S protein antibodies (higher than after the disease). Subsequent studies found that antibody levels decline rapidly with time after both vaccination and infection. Increased susceptibility to infection was also observed in the vaccinated group and those after infection. The kinetics of antibody titers after vaccination may be of practical importance in developing vaccination programmes. The cellular immunogenicity of vaccines is also of interest to researchers. The role of T cells in response to vaccination is not well understood but is thought to have a significant impact on vaccine efficacy and protection against infection [7]. Currently, one of the most pressing questions on everyone’s mind is the length/duration of protection after vaccination. The response to vaccination is individual and may depend on a number of individual and environmental factors and the vaccine formulation. Will there be a need for further doses of vaccination in the general population? Will it be recommended for specific groups? What is the most optimal time to administer it? Is it possible to establish antibody levels that protect against infection or severe COVID-19 course based on our current knowledge? It is still unclear whether the virus mutation is related to the lower vaccination efficacy or other factors (e.g., time since vaccination, choice of comparison groups) that influence the differences in the calculated rates [6,8]. 

This study aims to validate the complex (humoral and cellular) immune response over time in large population groups following mRNA vaccination (BNT162b2) of healthcare workers in real-life conditions and to provide an assessment of the relationship between antibody levels and T-cell activity in relation to the characteristics of the study group, including the identification of factors associated with vaccine immunogenicity and the persistence of long-term (humoral and cellular) responses to vaccination.

## 2. Materials and Methods

The study, which began in November 2020, involved 732 medical personnel from two hospitals (*n* = 460 + 272). Of these, 344 received the full vaccination and had blood drawn three times. They were vaccinated with Pfizer-BioNTech’s BNT162b2. The first dose was administered in December 2020/January/February 2021 (25 December 2020–10 February 2021), and the second dose January/February/March (17 January–8 March 2021). The median interval between vaccinations was 21 days (IQR: 21–21). 

Antibody assays were performed before vaccination, then between 22 February and 31 March 2021 (the number of days since the second dose of vaccination, the median 49, IQR: 42–57), and before the third dose 25 August–29 September 2021 (number of days the median 230, IQR: 224–241.5). 

The healthcare workers group was divided according to the nature of their work into the following categories: doctors, nurses/paramedics, physiotherapists, care managers, room attendants, administrative staff, laboratory staff.

The kinetics of the response to vaccination was examined by the titres of anti-SARS-CoV-2 IgG antibodies against the S1 protein (S1 subunit of the S protein) located within the virus spike in blood samples collected on three occasions. Antibody levels before vaccination were indicated as IgGT0, 4–9 weeks after full vaccination as IgGT1, and approximately 7–9 months after vaccination (before the booster dose) as IgGT2. The cellular response was tested once in a group of employees of one hospital (*n* = 189) in the third blood draw with the Quan-T-Cell SARS-CoV-2 IGRA assay from Euroimmun.

Humoral immune responses were measured with the anti-SARS-CoV-2 QuantiVac ELISA (IgG), which allows precise quantitative testing of IgG class neutralising antibodies directed against the S1 protein of SARS-CoV-2 virus (compatibility with neutralisation tests). The results are given in standardised international units: BAU/mL (BAU = Binding Antibody Unit). The test is used to assess the immune response to vaccination and after infection with the SARS-CoV-2 virus. Tests were performed following the manufacturer’s instructions. As per manufactured recommendations, antibody levels above 35.2 BAU are considered a positive result. 

Cellular immune response measurements were performed with a EUROIMMUN QuanT-Cell SARS-CoV-2 (IGRA) assay, which is used to assess the activity of T cells stimulated by a pathogen presence. The T cells present in the blood of a person who has been in contact with the virus or received vaccination can recognise the virus antigens. The test involves the in vitro stimulation of T cells with the S1 subunit of the SARS-CoV-2 virus spike protein. Activated T cells synthesise and release IFN-γ. Quantitative assessment of secreted IFN-γ is performed by ELISA. Tests were performed following the manufacturer’s instructions. As per manufactured recommendations, antibody levels above 200 mIU/mL are considered a positive result. 

Data on health status and lifestyle are based on surveys conducted among the subjects.

Adverse effects after the first and second dose of vaccination were assessed in separate surveys, including the occurrence of local (i.e., redness, swelling, pain) and general (fever, fatigue, headache, chills, vomiting, diarrhoea, myalgia, arthralgia) reactions, and grading of severity on a scale of 0: none, 1: mild, 2: moderate 3: severe, 4: very severe, based on the Food and Drug Administration’s guidance for toxicity grading scales for vaccines [9].

This study received approval from an independent ethics committee (No. KB 634/2020) and fulfilled the ethical guidelines of the Declaration of Helsinki. All study participants gave written informed consent before enrolment.

Statistical analysis was performed using R 3.6.1 of the R Project for Statistical Computing, Vienna, Austria. Categorical variables were presented as frequencies with percentages, whereas median with interquartile range (IQR) or mean with standard deviation (SD) were used to describe continuous variables. Categorical variables were compared using χ^2^ and Fisher’s exact test. Evaluation of data normality was performed using the Shapiro–Wilk test. Non-normally distributed continuous variables were compared using the Mann–Whitney–Wilcoxon test. Multiple comparisons were made with the Kruskal–Wallis test, post-hoc Dunn test, and multivariate ANOVA (MANOVA) and logistic regression. Natural logarithms of applied values were calculated and compared using parametric tests (*t*-test and ANOVA) to examine immunoglobulin level changes. *p*-value was considered statistically significant.

## 3. Results

### 3.1. Study Group

The study population consisted of 344 staff members of two hospitals who received the full vaccination and from whom three blood samples were collected at the following intervals: before vaccination, 4–9 weeks after vaccination, and 7–9 months after vaccination. 

The mean age of the subjects was 49 ± 11 years, and the median was 50. The youngest subject was 22, and the oldest was 72 (Table 1). Women accounted for 82% (*n* = 281). Chronic diseases were reported in 108 out of 344 (31%) participants.

Amongst the subjects, the largest group were nurses/care managers/paramedics, 44% (*n* = 153); non-surgeons, 21% (*n* = 71); administrative staff, 10% (*n* = 33); laboratory staff, 15% (*n* = 51); surgeons, 5% (*n* = 17); in addition to physiotherapists, ward attendants, and stretcher-bearers.

One hundred fifty-three individuals who had undergone infection before vaccination were identified by a positive nasopharyngeal swab by PCR and/or a positive anti-SARS-CoV-2 IgG or IgM serological test performed before vaccination when available. A positive result (above the reference value) for IgGT0 antibodies before vaccination was reported in 119 subjects. In the study group, 191 out of 344 (55.5%) subjects did not present past SARS-CoV-2 infection.

### 3.2. Humoral and Cellular Response to Vaccination

In the assay after full vaccination (i.e., after two doses), all subjects had positive results for anti-SARS-CoV-2 IgGT1 antibodies according to the manufacturer’s reference value (35.2 BAU/mL). The median antibody level after full IgGT1 vaccination was 2511.0 BAU/mL (interquartile range-IQR: 1518.00–4455.75), min. 90.57, max. 18,665. In a blood sample taken before the third dose (7–9 months after the second one), the median anti-SARS-CoV-2 IgGT2 antibody level was 261.8 BAU/mL (IQR: 157.66–732.75), min. 35.23, max. 8960. All results were also positive in this assay, but the values were lower by more than 9.5 times. The lowest values were close to the cut-off point.

**Figure 1:**
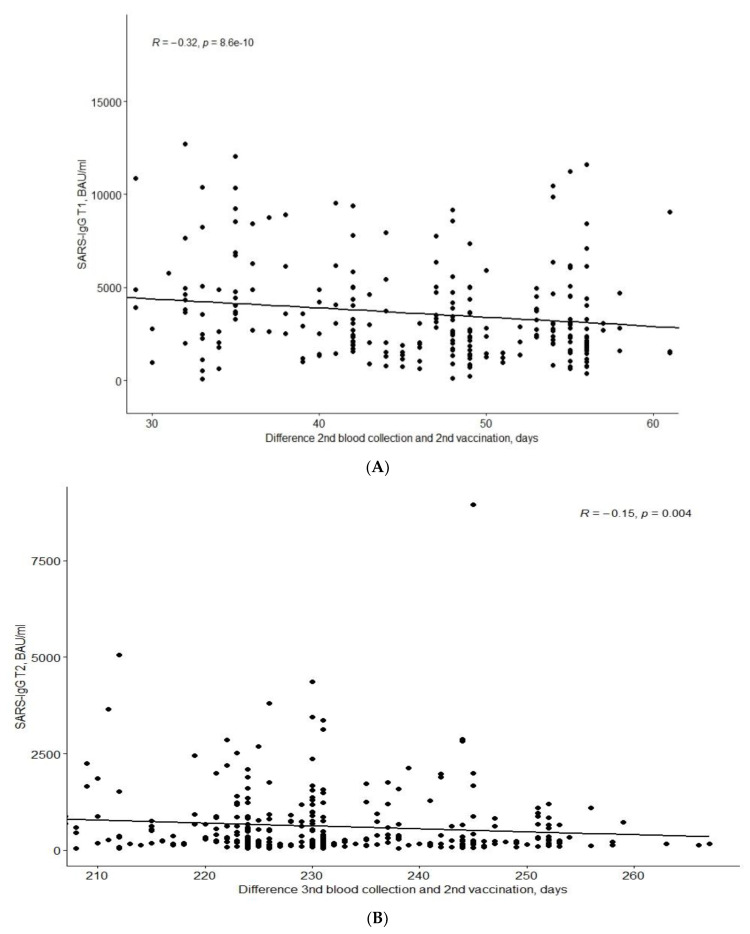
Time dependence (**A**) of IgGT1 titre since the second vaccination dose; (**B**) of IgGT2 titre since the second vaccination dose.

As expected, the titre of antibodies decreased in time from the second dose (Figure 1).

The level of serological response to IgGT1, IgGT2 vaccination was not correlated with age, gender, BMI, blood group, chronic diseases, smoking, physical activity, or influenza vaccination.

The cellular response was tested in 189 subjects, median: 1223.1 mIU/mL (IQR 487.33–3342.61), min. 0.5; max. 65,617, and 12 subjects had negative results based on the manufacturer’s standard (<200 mIU/mL).

There was a statistically significant difference in cellular response (based on QuanT-Cell result) according to gender (*p* = 0.02). In women, the median QuanT-Cell level was 1120.52 mIU/mL (IQR 540.59–2725.39), while in men, 2677.58 mIU/mL (IQR 703.96–5649.12).

There was a positive trend between the level of cellular response and BMI (*p* = 0.062—borderline result). Overweight subjects had a higher QuanT-Cell response rate, with a median of 1486.09 mIU/mL (IQR 709.23–4444.45), while those with a normal BMI had 1161.83 mIU/mL (IQR 357.39–3036.37).

Higher responses in the T-cell activity test were reported in persons who had an infection (*p* = 0.0018), median QuanTcell: 1775.81 mIU/mL (IQR: 786.2–5474.88) vs. 951.8 mIU/mL (IQR 381–2534.95) in those with and without past SARS-CoV-2 infection, respectively. There was no significant difference in cellular response in the SARS-CoV-2 infection severity analysis.

There was no correlation of T cell activity with age, blood group, chronic diseases, smoking, physical activity, influenza vaccination, and vaccine adverse events (VAEs). 

**Figure 2:**
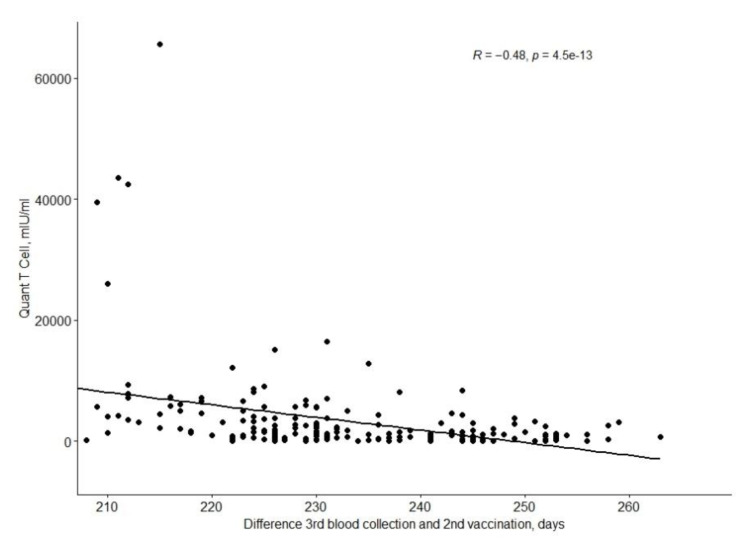
Time dependency of QuanT-Cell response since the second vaccination dose.

The QuanTCell response decreases with time after full vaccination, as shown in the Figure 2.

QuantCell values depending on the time since the second vaccination. 

**Figure 3:**
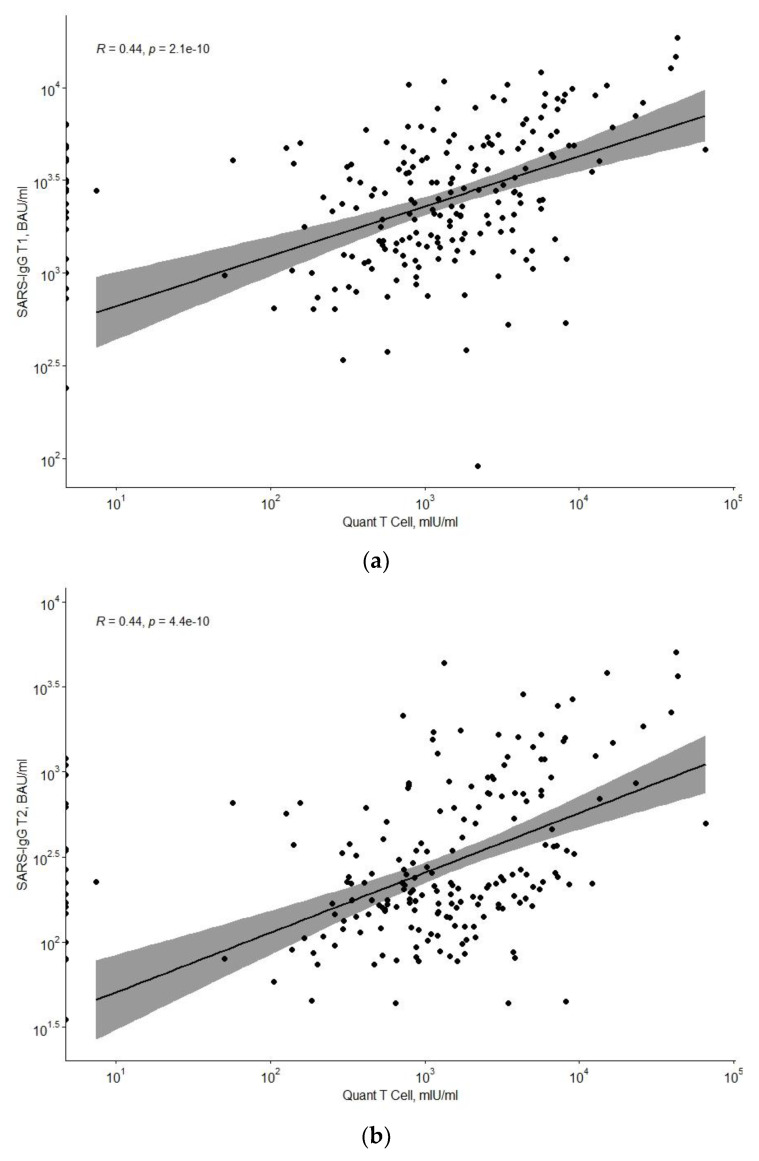
Dependence of QuantCell levels on IgGT1 (**a**) and IgGT2 (**b**) concentrations.

QuantCell levels correlate statistically significantly with IgGT1 (positive correlation, with a mean R-score of 0.44; *p* < 0.001) and IgGT2 (positive correlation, with a mean R-score of 0.44; *p* < 0.001) (Figure 3).

There were no statistically significant differences between the positive and negative QuantT-Cell groups in terms of age, gender, BMI, chronic diseases, blood group, and VAE. 

It was shown that eight out of 12 persons (67%) with a negative QuanT-Cell result were smokers (*p* = 0.014).

### 3.3. Humoral and Cellular Responses and Past SARS-CoV-2 Virus Infection

Subjects without past infection had significantly lower anti-SARS-CoV2 IgGT1 antibody titres compared to those with confirmed infections. Median IgGT1 antibody titres were 1922 BAU/mL (IQR 1193–3105) vs. 3597 BAU/mL (IQR 2151–5557.75), respectively (*p* < 0.05). The value in persons with a history of infection before vaccination was almost twice as high.

For assays at 7–9 months, the statistically significant differences in antiSARS-CoV-2 IgGT2 antibody levels persisted. The median for those without a past infection was 178.02 BAU/mL (IQR 110.76–283.99), while those with confirmed infection, 663 BAU/mL (IQR 295.3–1303.25) (*p* < 0.05), which was 3.7 times higher.

**Figure 4:**
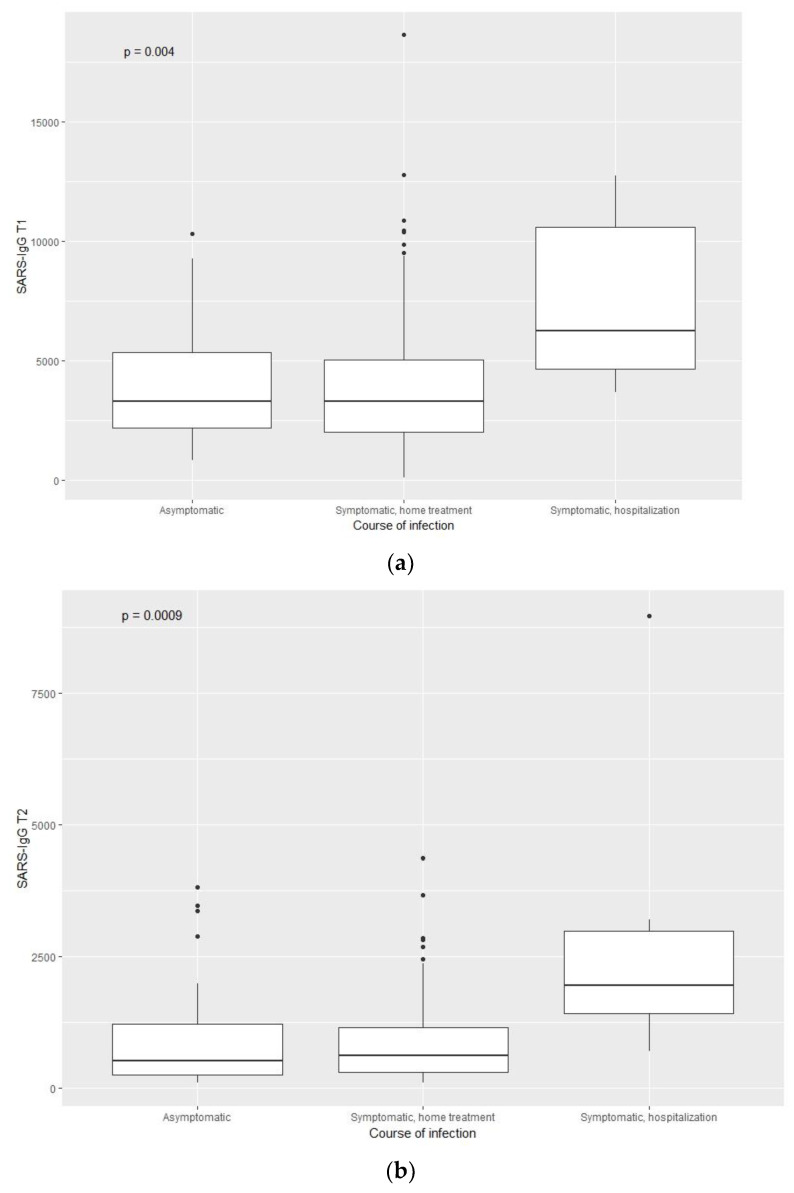
Dependence of anty-SARS-CoV-2 IgGT1 (**a**) and anty-SARS-CoV-2 IgGT2 (**b**) titre and the course of infection.

Furthermore, a correlation was found between the course of infection (asymptomatic, symptomatic, home treatment; symptomatic, hospitalisation) and IgGT1 and IgGT2 antibody titres (Figure 4).

In a study of the relationship of the decrease in antibody titre with IgGT1 to IgGT2 values, no association was found with age, gender, BMI, and chronic diseases.

### 3.4. Decrease in Antibodies over Time (IgGT1–IgGT2 Difference)

Between the second and third collection, median days 182 (min.121–max.228, IQR: 170–189), there was a more than nine-fold decrease in antibody titres (median anti-SARS-CoV-2 IgGT1: 2511.0 vs. median anti-SARS-CoV-2 IgGT2: 261.8). There was no association of antibody decline with age, gender, BMI, blood group, chronic diseases, smoking, physical activity, or VAEs. Based on statistical analysis, there was a significantly lower decrease in titers of antibodies in persons with past SARS-CoV-2 infection, but no correlation with the severity of the infection course. In those without past infection, the median level of the logarithmic antibody difference was −2.31 (−2.74 to −1.94), and in those with past infection, it was −1.69 (−2.15 to −1.30).

### 3.5. Vaccine Adverse Events (VAEs) and the Immune Response to Vaccination 

**Figure 5:**
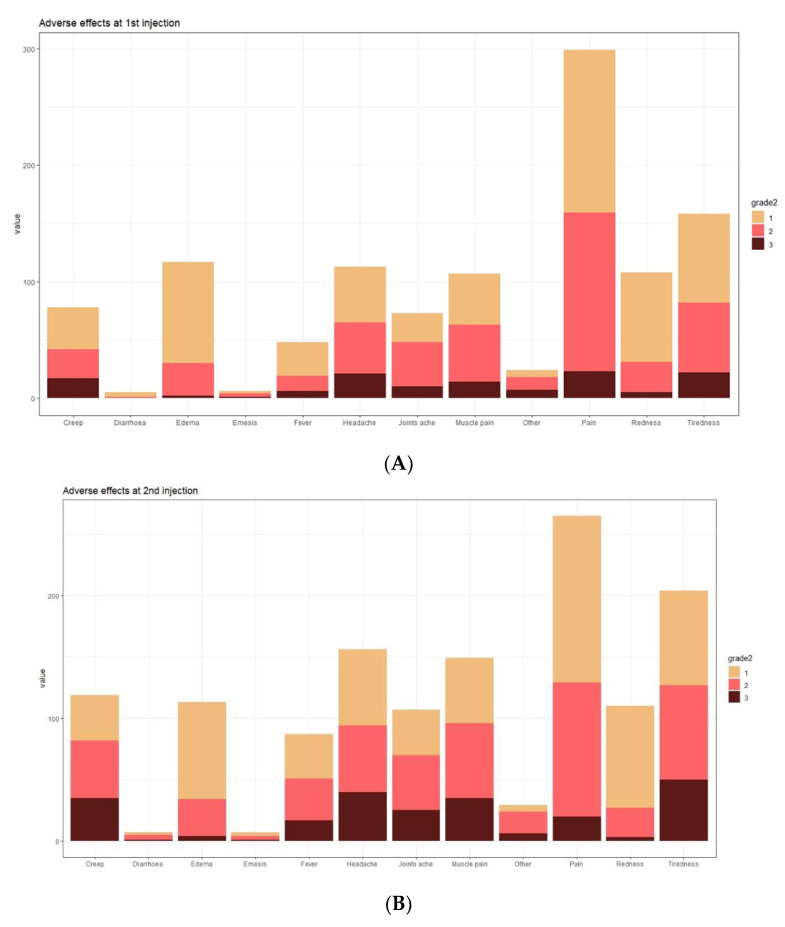
Systemic and local adverse events after first (**A**) and second (**B**) injections according to the grade of intensity.

In the analysed group, vaccine adverse events (VAEs) after the first dose of COVID-19 vaccine were observed in 95% of subjects (327 out of 344), while after the second dose in 89% (309 out of 344). The predominant adverse events (regardless of severity) were local pain (89.5%, 79.5%), oedema (35.3%, 34.7%), and redness at the injection site (32.5%, 33.6%) (frequency after the first and second dose, respectively). The most commonly reported systemic symptoms were fatigue (48.3%, 61.5%), headache (34.5%, 47.3%), and myalgia (32.2%, 45.0%) (frequency after the first and second dose, respectively) (Figure 5).

After the first dose of the vaccine, mild (0–5 points), moderate (6–10 points) or severe VAEs (>10 points) were observed in 67%, 17%, and 16% of subjects, respectively, and after the second dose of the vaccine in 52%, 24%, and 24% of subjects, respectively. The median severity of VAEs after the first dose of vaccine was 3.00 (IQR: 2.00–7.00). After the second dose of vaccine, the median was 5.00 (IQR: 2.00–10.00). There were no severe VAE cases complicated by anaphylactic shock in the analysed group, requiring hospitalisation or leading to death. 

There was no association between the VAE severity after the first dose of COVID-19 vaccination and anti-SARS-CoV-2 IgGT1 (*p* = 0.34) and anti-SARS-CoV-2 IgGT2 (*p* = 0.206) antibody titres.

There was a correlation between the VAE severity after the second dose of vaccine and the anti-SARS-CoV-2 IgGT1 antibody titre (*p* = 0.015). Subjects with moderate and severe VAEs had a greater humoral response (higher antibody titres) compared to subjects with minor VAEs—median antibody titres, respectively: 2989.0 BAU/mL (IQR: 1518.00–5008.65) (*p* = 0.035), 2805.50 BAU/mL (IQR: 1590.25–4870.50) (*p* = 0.023), 2163.00 BAU/mL (IQR: 1424.00–3911.38)—for VAEs of 6–10 points, >10 points and 0–5 points.

Subjects with severe VAE symptoms after the second dose of vaccine, compared to subjects with mild VAEs, had higher titres of anti-SARS-CoV-2 IgGT2 antibodies (*p* = 0.06, borderline); median titre for VAEs > 10 points 348.32 BAU/mL (IQR: 162.22–890.75) vs. VAEs of 0–5 points 239.0 BAU/mL (IQR: 147.7–3651.00), respectively.

Analysing the association of specific VAEs with vaccine response, a statistically significant correlation was found between the severity of fever and chills after the first and second vaccine doses and IgGT1 and IgGT2 antibody titres (*p* < 0.001). Furthermore, a correlation was observed between muscle pain and IgGT1 and IgGT2 titres (*p* < 0.01).

There was no difference between the VAE severity after the first and second vaccine doses and the decrease in antibody titres (logarithmic lnIgGT2–InIgGT1 antibody difference) (*p* = 0.32 and *p* = 0.6, respectively).

The VAE severity did not correlate with the cellular response to vaccination.

### 3.6. SARS-CoV-2 Infections following Vaccine Administration

We recorded SARS-CoV-2 infection in six persons after vaccine administration. In two, the infection occurred after the first vaccine dose; in one, the virus was identified five days after the second dose. The history shows that the infection occurred after the first vaccination.

Patient data are shown in the Table 2.

## 4. Discussion

BNT162b2 is the first mRNA vaccine used on such a large scale, so its immunogenicity is not yet fully understood. The results of our real-life observation among medical personnel demonstrate a good safety profile, high comprehensive immune response to complete vaccination with two doses of Pfizer-BioNTech’s BNT162b2, and confirm data from randomised clinical trials [5] and other observational studies, especially those among medical personnel [10,11]. 

Measurement of the antibodies titre by ELISA is a simple, globally widespread, and relatively inexpensive method for assessing the post-vaccination response to the SARS-CoV-2 virus. In all medical personnel after full vaccination, we found a positive result (above the accepted cut-off point) for anti-SARS-CoV-2 IgG antibodies (EUROIMMUN QuantiVac ELISA) in both the first sample collected at 4–9 weeks (anti-SARS-CoV-2 IgGT1) and the second one at 7–9 months after full vaccination (anti-SARS-CoV-2 IgGT2). We demonstrated that the humoral response after vaccination was maintained in vaccinated hospital staff more than six months later (7–9 months after the second dose). The persistence of IgG antibodies for months after full vaccination in a significant proportion of individuals in selected populations of medical personnel has also been described in previous papers [12,13,14,15]. 

Convincing data show a poorer humoral response in men, obese individuals and the elderly [11,15]. However, as in our study, some researchers do not note such a correlation [10,16,17]. Differences due to gender or obesity tend to be minimal in a healthy population [16]. On the other hand, the lack of correlation with age in our group can also be explained by good health and a small proportion of older individuals, particularly ones over 65.

A crucial factor affecting anti-SARS-Cov-2 IgG antibody titre is past SARS-CoV2 infection [14,18,19,20]. In the study group, past infection and severity of infection symptoms correlated positively with the immune response to vaccination. The antibody titre in the 4–9-week assay was almost twice as high in individuals with past infection and 3.7 times higher in the subsequent 7–9-month assay than in individuals without SARS-CoV-2 infection history. 

The phenomenon of a stronger response to vaccination in individuals with past infection may be explained, among other things, by the fact that recovery from infection may induce an immune response directed against various antigens of the virus and not just the spike protein as in the case of vaccination. 

Ferrari et al. showed that past SARS-Cov-2 infection provides an immunological memory that persists for many months. The administration of one vaccination dose in persons with COVID history acts as a ‘booster’ and causes a stronger humoral immune response (an antibody titre approximately 10 times higher than in persons without COVID history). It was shown that even persons with low antibody titres after being ill are able to mount a strong immune response after a single dose of the vaccine [21]. However, this study did not assess the cellular response, which may also contribute significantly to resistance to subsequent infection. Manisty et al. compared the immune response, post-infection and post-vaccination, by measuring antibodies to S protein in subjects who had previously naturally recovered from COVID and subjects who had not been infected. The post-infection response was comparable to the post-vaccination response after one dose in persons without past SARS CoV-2 infection. In contrast, administering a single dose of the vaccine in persons who had survived COVID resulted in them having anti-SARS-CoV-2 antibody titres up to 140 times higher 21 days after the first dose of anti-SARS-CoV-2 than before vaccination. On the contrary, among individuals without past infection, the response 21 days after the first dose of vaccination gave significantly lower antibody levels [22]. 

Ali Ahmad et al. examined the humoral response in more than 1000 persons vaccinated against SARS-CoV-2 by assessing the levels of anti-S1 antibodies in IgG, IgM and IgA and neutralising antibodies [23]. Comparisons were made between those vaccinated with two doses and those vaccinated with one dose and COVID-19 history. It was found that people who had previously undergone COVID-19 had a significantly better response to vaccination, with antibodies persisting considerably longer than those who had not. Ferrari et al. and Manisty et al. investigated differences in the post-vaccination response in people who had previously had COVID and suggested using different vaccination schedules in individuals with past infection [21,22]. Krammer et al. and Mak et al. additionally showed that in persons with past SARS-CoV-2 infection (even more than a year before vaccination), the administration of one vaccine dose produces the same humoral and cellular response as administration of two vaccine doses [4,24]. 

In the context of the influence of past infection on vaccine immunogenicity, the relationship with the disease course should be emphasised. A positive correlation of the symptom severity of SARS-CoV-2 infection with the antibody titre was also demonstrated by Wolszczak-Biedrzycka [14]. Although past infection is associated with preserving immunological memory even one year after infection [4], it can be expected to be weaker in certain groups. This includes the elderly, residents and staff of care homes, persons after paucisymptomatic infection and those with higher exposure [25]. The risk of recurrence was higher in older individuals, care home residents, and persons after asymptomatic COVID infection. Many studies suggest that the immune response in persons after asymptomatic COVID infection or with mild symptoms is weaker [25].

In our group, we did not observe any association of vaccination response with chronic diseases. Other authors are yet to describe an association between thyroid disease and immune response after vaccination or an association between hyperglycaemia, diabetes, and the kinetics and persistence of neutralising antibodies against the SARS-CoV-2 spike protein [26].

As expected, we observed a decrease in antibody titres over time. Like Olariu et al., we noted a reduction in antibodies by as much as 90% (median anty-SARS-CoV-2 IgGT1 antibodies: 2511.0 vs. anty-SARS-CoV-2 IgGT2 antibodies 261.8) over 7–9 months [13]. On the other hand, Ferrari has achieved a drop of 70% after six months [12]. Despite such a significant decrease in antibody levels, it is important to emphasise the persistence of a significant difference in antibody titres between those without and with a history of infection—178.02 (IQR 110.76–283.99) vs. 663 (IQR 295.3–1303.25) which, when consistent with data from multiple observational studies, may provide a serious argument in the discussion about individualising vaccination schedules [12,14].

Cellular immunological response to vaccination after 7–9 months was achieved in more than 93% of the study group. Male gender was associated with higher levels of cellular response. In addition, higher Quan-T-Cell values were noted in overweight subjects (borderline results). Choi evaluated the cellular response to a vector vaccine, which generally shows greater cellular immunogenicity than mRNA vaccination [27]. Similarly to our population, higher levels of cellular response were found in men. On the other hand, the correlations related to BMI were divergent: in Choi, with an increase in BMI, a worse cellular response was recorded, while in our group, higher values were found in overweight subjects (borderline results). In the above study, there was no correlation of smoking with the level of cellular response to vaccination. Smoking is not often analysed in the context of vaccine response, although an association of severe COVID-19 with smoking was shown. Noteworthy, in our study, the lack of cellular response mostly concerned smokers, whereas Herzberg and Watanabe showed an association of smoking with a weaker humoral response [10,28]. As in the humoral response, an important factor influencing the Quan-T-Cell result is a history of infection, as also confirmed by Zollner and Prendecki [20,29].

Also of interest is the association of VAEs with the immune response to vaccination. VAEs are frequently reported in COVID-19 vaccinated individuals, regardless of the type of vaccine, but in most cases, they involve local adverse reactions of mild severity and short duration [30]. According to data relating to the UK population, the incidence of local VAE was 71.9% after the first dose of mRNA vaccine (BNT162b2, Pfizer-BioNTech) and 68.5% after the second dose (for general symptoms: 13.5% and 22%, respectively) [31]. In the analysed population of medical personnel, the incidence of vaccine adverse events (BNT162b2) after the first dose was 92% and after the second dose, 88% (overall data for all VAEs), which is consistent with previous reports. Analysis of the literature indicates that the severity of local and general vaccine adverse events does not significantly affect the humoral response after vaccination with either vector or mRNA vaccines [10,32,33,34]. On the other hand, however, some reports highlight a possible link between VAEs and the degree of the immune response. Choi et al. found no overall effect of VAEs, including severity and duration of symptoms, on humoral or cellular responses in their study evaluating the relationship between reactogenicity and immunogenicity following vector vaccine administration [27]. However, analysis of individual local and general vaccine adverse events showed an association of erythema (also local pain, a weaker correlation here) after the first dose with neutralising antibody titres and chills after the second dose with anti-SARS-CoV-2 S1 IgG and neutralising antibody titres. Park et al. showed a higher incidence of general post-vaccination symptoms and a greater need for antipyretics in the group of subjects with a confirmed immune response in the form of anti-S1 antibody synthesis to the first dose of vector vaccine against COVID-19 [35]. Our results also indicate that reactogenicity may be important for the immune response. The severity of some systemic VAEs (fever, chills) correlated with the humoral response in the short and long term. Subjects with moderate (*p* = 0.035) and severe VAEs (*p* = 0.023) after the second dose of the vaccine had higher antibody titres than those with minor VAEs in short- and long-term follow-up. However, only borderline significance was achieved in assessing antibody titres 7–9 months after the second dose (*p* = 0.6). However, we did not confirm the association of VAEs with the cellular response. The relationship of VAE severity to response to vaccination remains unclear and requires further study. However, our results suggest that reactogenicity may be an indicator of immune response to vaccination.

This analysis of the immune response to vaccination also has a practical context. There were six confirmed cases of SARS-CoV-2 infection in the study group in the period 7–9 months after vaccination. Three occurred after the first dose and three more after full vaccination. The course of the infections was mild. Tré-Hardy found infection in only one out of 201 medical personnel at 6-month follow-up; Ferrari reported the same in 12 out of 1054 persons [12,19]. ‘Breakthrough infections’ after vaccination are rare. However, no vaccine is 100% effective, so it is important to vaccinate at high rates in specific populations. Interestingly, the infection can occur even when there is a good response to vaccination [12].

However, the relationship between antibody concentration and the infection rate is straightforward. Analyses of vaccine efficacy data in large populations show that efficacy decreases with time after vaccination, even when virus variability is considered [6]. The efficacy of BNT162b2 vaccination estimated at around 93–97% in the first month may drop to approximately 53–67% after about 4–5 months. The possibility of the emergence of different virus variants also needs to be considered here [6]. Compared to these figures, the infections we record among hospital staff with high exposure to the virus seem decidedly rare. This may be explained by the high proportion of persons with the past infection before vaccination. There may also be a significant proportion of the study group who have had a clinically silent infection following vaccination.

The decrease of the antibody titre was the reason for introducing the third vaccination dose. The determination of protective antibody levels against infection/severe disease is still the subject of ongoing research [6,8,10,36]. Despite the high dose of antibodies, infections occur incidentally as breakthrough infections. We still do not know whether there are antibody levels that truly protect against infection. In his analysis, Kertes set a protective level of 300 AU/mL (Abbott test, 6× above the cut-off point) [8]. The lowest percentage of the infected was observed for values above or equal to 800 AU/mL. The Feng study presents a very practical approach to this problem [36]. Data from clinical trials of the vector vaccine by ChAdOx1 nCoV-19 were analyzed for the relationship of the level of individual humoral response to vaccination at 28 days after the second dose with a risk of symptomatic infection over a further 6 months, thus determining threshold values with 264 BAU/mL for anti-spike antibodies. Of course, virus variability over time and subsequent mutations, which may reduce the effectiveness of currently available vaccines, must be taken into account.

The decreasing humoral response to vaccination raises the question of the level of cellular response, which in our study was assessed in parallel with the determination of anti-SARS-CoV-2 IgG3 antibodies. Cellular and humoral responses show a moderate positive correlation. However, especially in the graphical representation, it can be seen that there are those with a high cellular and low humoral response among the vaccinated individuals and the opposite with a high humoral and low cellular response. For the time being, it is difficult to explain these discrepancies, which researchers still study [4,37].

Our study was performed in a day-to-day practice setting in a population of hospital staff; due to the ongoing pandemic, coordinated blood collection and vaccination was possible. In addition, health and lifestyle data were obtained from the subjects. However, it cannot be excluded that individuals with poorer health status, multiple chronic diseases, autoinflammatory diseases, and immunosuppressive treatment postponed the vaccination, and our group was therefore not fully representative. In Poland, vaccination has been mandatory for medical personnel since 1 March 2022. Due to the highly dynamic nature of humoral and cellular responses over time, the exact interval between vaccination administration and sample collection may have been important in the statistical analysis of the relationships studied. Data on health status, vaccination, SARS-CoV-2 infection, and post-vaccination symptoms were derived from questionnaires completed by patients. Medications taken were not analysed. This may have influenced the underestimation of reported comorbidities with a significant impact on the response to vaccination [38,39,40].

The study results indicate that mRNA vaccination BNT162b2 is safe and effective until about 7–9 months.

Understanding the immune response to COVID-19 vaccination is key to developing effective preventive measures during this pandemic. Comprehensive immune response studies derived from real-life practice in large cohorts can make an important contribution to our knowledge. There are few such data in the literature to date. Identifying critical factors that significantly determine the response to vaccination will enable the introduction of an appropriate strategy to implement the vaccination programme.

## Figures and Tables

**Table 1:** Characteristics of the participants in this study.

	Total, *N* = 344 No. (%)
Gender	
Male	63 (18)
Female	281 (82)
Age median (min.–max.)	50 (22–72)
BMI	
Overweight	186 (54)
Normal	150 (44)
SARS-CoV-2 infection	
None	192 (56)
Asymptomatic	35 (10)
Symptomatic, home treatment	108 (31)
Symptomatic, hospitalisation	10 (3)
Subject category	
Administration	33 (10)
Physiotherapist	8 (2)
Non-surgeon	71 (21)
Surgeon	17 (5)
Nurse/paramedic/care manager	153 (44)
Laboratory assistant/technician/pharmacist	51 (15)
Salaries/stretcher-bearers	11 (3)
Smoking	
No	282 (83)
Yes	58 (17)
Blood type	
0	106 (35)
A	112 (36)
B	67 (22)
AB	25 (8)
VAE 1	
0–5	229 (67)
6–10	59 (17)
>10	56 (16)
VAE 2	
0–5	178 (52)
6–10	84 (24)
>10	82 (24)
Chronic diseases	108 (31)
Renal diseases	2 (1)
Cardiovascular diseases	35 (10)
Lung diseases	7 (2)
Rheumatic diseases	4 (1)
Neurological diseases	5 (1)
Hashimoto’s disease	31 (9)
Diabetes	4 (1)
Influenza vaccination (data from one hospital *n* = 203)	118 (58)
Physical activity	146 (42)

BMI: body mass index, VAE 1: vaccine adverse events after 1-st dose of the vaccine, VAE 2: vaccine adverse events after 2-nd dose of the vaccine.

**Table 2:** Characteristic of persons with SARS-CoV-2 infections following vaccine administration.

No./Initials	1/EW	2/KW	3/BS	4/MT	5/AJ	6/KN
Age/years	30	62	46	54	44	52
Gender	F	F	F	M	F	M
Subject category	Non-surgeon	Non-surgeon	Non-surgeon	Laboratory staff	Nurse	Surgeon
Confirmed infections	PCR test	PCR test	PCR test	PCR test	PCR test	PCR test
Infection course	Asymptomatic	Symptomatic, home treatment	Symptomatic, home treatment	Symptomatic, home treatment	Symptomatic, home treatment	Symptomatic, home treatment
Comorbidities	Hashimoto’s disease	Allergy	Cardiovascular disease	None	None	Cardiovascular disease Hypertension
Smoking	Never smoked	Never smoked	Former smoker	Never smoked	YES	Never smoked
VAE-1	2	9	3	6	11	3
VAE-2	1	10	8	6	3	0
SARS-IgG1	0.57	0.06	0.09	0.06	0.05	0.09
SARS-IgG2	4105	1578	2103	1857	104.79	2486
SARS-IgG3	248.78	660	1151	3613	460	143.07
Quan-T-Cell	1515.8	1377.6	5459	2752.6	Not determined	Not determined
Time of infection in relation to vaccination * after first vaccination; ** after second vaccination	14 *	49 **	68 **	76 **	15 *	5 **

## Data Availability

The data that support the findings of this study are available on reasonable request from corresponding author.
